# Molecular basis for adaptive evolution of aromatic degradation enzymes in bacteria revealed by metagenomics

**DOI:** 10.3389/fmicb.2026.1795400

**Published:** 2026-04-13

**Authors:** Hikaru Suenaga, Hidehiko Fujihara

**Affiliations:** 1 Cellular and Molecular Biotechnology Research Institute, National Institute of Advanced Industrial Science and Technology (AIST), Tsukuba, Japan; 2 Department of Food Management, Faculty of Nutrition Sciences, Nakamura Gakuen University, Fukuoka, Japan

**Keywords:** adaptive evolution, aromatic compounds degradation enzymes, biocatalysis, bioremediation, metagenome

## Abstract

Aromatic hydrocarbons, including persistent polycyclic aromatic hydrocarbons (PAHs), impose strong selective pressures that drive the adaptive evolution of bacterial degradation systems. Metagenomic studies have revealed extensive diversification of key catabolic enzymes, such as ring-hydroxylating and ring-cleavage dioxygenases, through the accumulation of single-nucleotide polymorphisms (SNPs) and structural modifications that increase substrate range and enhance catalytic efficiency in polluted environments. These findings demonstrate that gene mutations that change enzyme properties collectively shape the evolution of aromatic-degrading bacteria. Metagenomics is powerful tools for elucidating these evolutionary processes and advancing applications in bioremediation and industrial biocatalysis.

## Introduction

Industrial development, the use of agrochemicals, and other anthropogenic activities have resulted in the release of hazardous aromatic compounds, including polycyclic aromatic hydrocarbons (PAHs), into the environment (Nagy et al., 2024). PAHs are a class of persistent organic pollutants produced by both natural and anthropogenic processes and are widely detected in soils and sediments worldwide. Due to their pronounced hydrophobicity and chemical stability, these compounds tend to undergo adsorption onto suspended particles and ultimately accumulate as sediment within aquatic ecosystems (Xu et al., 2015; Fang et al., 2020). This results in the accumulation of high concentrations of aromatic compounds in the sediment where they pose significant risks to living organisms (Fuchs et al., 2011). There is therefore a great deal of interest in mechanisms of degradation of aromatic compounds and remediation of contaminated sediments (Furukawa and Fujihara, 2008).

Despite their toxicity and persistence, many environmental microorganisms can exploit aromatic compounds as sources of carbon and energy (Ghosal et al., 2016; Fujihara et al., 2023). Microorganisms possess an exceptional capacity for rapid adaptation to novel or fluctuating environmental conditions. Elevated concentrations of aromatic compounds eliminate susceptible microorganisms, whereas others adapt and survive under these selective pressures. As enzymes are the fundamental unit of metabolism in all living organisms, genes encoding degradative enzymes play a crucial role in microbial adaptation. Investigating enzyme evolution requires comprehensive collections of related enzymes, as comparisons of sequence homology and parallel beneficial mutations within enzyme families provide valuable insights into how enzymes adapt to specific ecological contexts (Parales, 2003).

**Figure 1:**
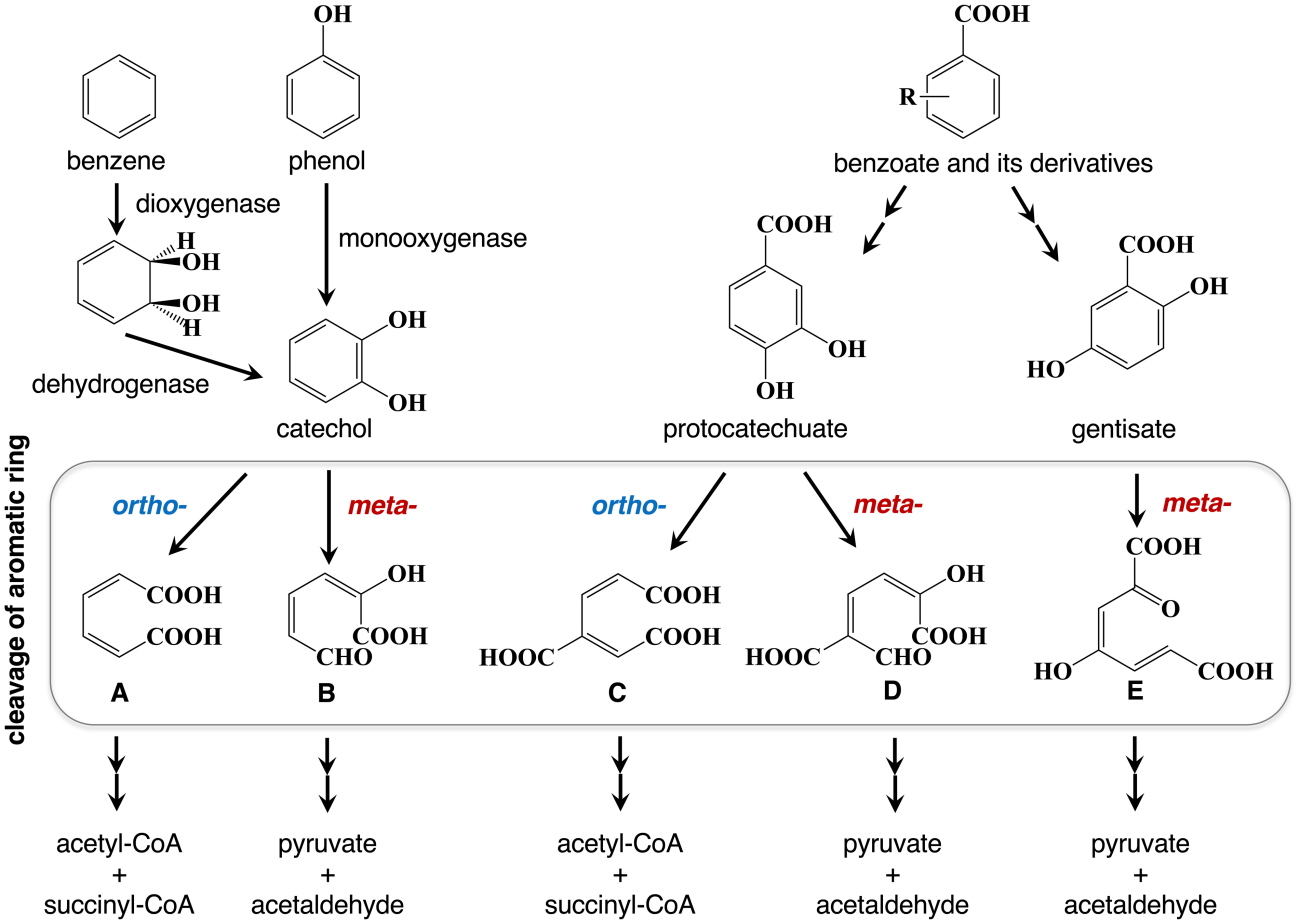
Bacterial aromatic compound degradation pathways. Bacteria metabolize aromatic compounds via dihydroxylated intermediates that undergo ring cleavage catalyzed by either intradiol or extradiol dioxygenases (*ortho*- or *meta*-cleavage). The resulting products are further processed into central metabolic intermediates. (A) *cis,cis*-moconate; (B) 2-hydroxymuconic semialdehyde; (C) 3-carboxy-*cis,cis*-muconic acid; (D) 2-hydroxymuconic semialdehyde-5-carboxylate; (E) maleylpyruvate.

Bacteria metabolize a remarkably broad spectrum of aromatic compounds including PAHs. Despite the considerable diversity of the degradation pathways, typical first step is the hydroxylation of an aromatic ring *via* a monooxygenase or dioxygenase, with the formation of a *cis*-dihydrodiol, which gets rearomatized to a diol intermediate by the reaction of a dehydrogenase (Figure 1). These pathways converge on a limited set of dihydroxylated intermediates, including catechol (1,2-dihydroxybenzene), protocatechuate (3,4-dihydroxybenzoate), and gentisate (2,5-dihydroxybenzoate) (Hirose et al., 1994; Hirano et al., 2007; Fetzner, 2012). Catechol and protocatechuate can undergo ring fission catalyzed by either intradiol or extradiol dioxygenases, corresponding to *ortho*- and *meta*-cleavage pathways. The *meta*-cleavage of catechol and protocatechuate yields 2-hydroxymuconic semialdehyde and 2-hydroxymuconic semialdehyde-5-carboxylate, respectively (Figure 1). The end products of these pathways are central metabolites that enter the tricarboxylic acid cycle (Ghosal et al., 2016). Accordingly, this review focuses ring-hydroxylating oxygenases and ring-cleaving dioxygenases, as the key enzymes of bacterial aromatic degradation.

Recent advances in culture-independent technologies, particularly metagenomics, have enabled the exploration of previously undercharacterized microbial communities and their roles in the degradation of aromatic compounds (Loviso et al., 2015; Hidalgo et al., 2020; Sandhu et al., 2022). Metagenomic analyses provide extensive sequence information on degradative genes, yielding critical insights into the adaptive evolution of aromatic degradation pathways. This review summarizes current understanding of the evolutionary mechanisms revealed through metagenomic approaches, with a particular emphasis on aromatic-degrading enzymes.

## Mutation-driven adaptation in aromatic degradation enzymes

Structure-oriented mutagenesis has repeatedly demonstrated that a small number of amino acid substitutions can change the substrate specificity as well as the regio- and enantioselectivity of ring-hydroxylating and ring-cleaving dioxygenases. In naphthalene dioxygenase (NDO) from *Pseudomonas* sp. strain NCIB 9816-4, substitution of a single active-site residue is sufficient to alter regio- and enantioselectivity on naphthalene and phenanthrene, whereas mutation of a single amino acid in the Fe-ligand abolishes activity (Parales et al., 2000a; Parales et al., 2000b). Changes of multiple amino acids predicted to interact with the substrate in the same NDO exhibited not only the alteration of regio- and enantioselectivity across naphthalene, biphenyl and phenanthrene but also a shift in the reaction mode among dioxygenation, monooxygenation and desaturation with indoline and 2-nitrotoluene as substrates (Yu et al., 2001). In 2-nitrotoluene 2,3-dioxygenase of *Acidovorax* sp. JS42, one amino acid substitution reshapes the binding pocket, playing a crucial role in both determination of enantiospecificity for naphthalene and ability to oxidize the ring of mononitrotoluenes (Lee et al., 2005). The gentisate 1,2-dioxygenases from *Corynebacterium glutamicum* oxidatively cleave the aromatic nucleus of gentisate (2,5-dihydroxybenzoate) but are not able to convert salicylate (2-hydroxybenzoate). Substitution of one amino acid that prevents the oxidation of (substituted) salicylate(s), as revealed by structural analysis, resulted in the acquisition of the ability to oxidize salicylate and several other monohydroxylated substrates (Eppinger and Stolz, 2017). Consequently, even minor amino acid substitutions in the vicinity of the substrate-binding site of the enzymes involved in aromatics degradation provide the host microorganism with the capacity to metabolize aromatic compounds that were previously impossible to metabolize. Such enzyme adaptations, based on amino acid substitutions caused by random mutations, are likely to occur in natural environments (Junca et al., 2004).

## Metagenomic approach for the comprehensive retrieval of genes for aromatic degradation enzymes existing in the environment

As described above, the adaptive evolution of aromatic ring-degrading enzymes under extreme conditions in the presence of aromatic compounds is accompanied by the accumulation of random mutations, among which variants providing functional advantages are thought to be the predominant group. Metagenomic analysis enables the comprehensive identification of enzyme variants that have accumulated random mutations under selective conditions. Functional characterization of these diverse variants provides a basis for inferring the molecular mechanisms and trajectories of adaptive evolution.

Gene-targeted metagenomics for biphenyl dioxygenase (aromatic ring-hydroxylating enzyme) based on PCR and pyrosequencing in polychlorinated biphenyl-contaminated soil has revealed three known conserved residues, as well as seven additional conserved residues (Iwai et al., 2010). The greater diversity revealed by this gene-targeted approach provides deeper insights into genes potentially important in environmental processes to better understand their ecology, functional differences and evolutionary origins (Zhou et al., 2006; Chemerys et al., 2014). Gene targeted metagenomic surveys of the PAH ring-hydroxylating dioxygenase across oilfield soils and mangrove sediments revealed that the spatial distribution of this gene was dependent on geographical location and regulated by local environmental variables, such as PAH contamination level, salinity, water content, pH and nutrient level (Liang et al., 2019).

A metagenomics-driven approach combined with hidden Markov models (HMMs) and bioinformatics predictions described in Szalkai and Grolmusz (2019) has been employed to identify novel PAH-degrading enzymes from soil metagenomes collected at a coal gasification plant site contaminated with PAHs (Meier et al., 2016; Nagy et al., 2024). Gentisate 1,2-dioxygenase is a key enzyme that catalyzes the *meta*-cleavage of gentisate (Figure 1). Candidate sequences corresponding to two gentisate 1,2-dioxygenase genes were selected based on novelty, structural integrity, crystallizability, and predicted heterologous expression in *Escherichia coli*. Three-dimensional modeling revealed conserved catalytic motifs alongside key sequence variations, including a Gly/Ala substitution pattern that influences substrate specificity in dioxygenases (Ferraroni et al., 2013). Soil microcosm experiments demonstrated that these enzymes substantially enhanced the degradation of low molecular weight PAHs, including naphthalene and phenanthrene, achieving up to 95% removal within 7 days. These findings highlight the effectiveness of metagenomic mining in uncovering PAH-degrading enzymes that are well adapted to harsh environmental conditions.

Extradiol dioxygenases (EDOs) are also key enzymes in aerobic aromatic degradation pathways, catalyzing the *meta*-cleavage of catecholic and protocatechuate intermediates (Brennerova et al., 2009) (Figure 1). Functional metagenomic analyses of biostimulated petroleum-contaminated soils in three different studies have revealed exceptional diversity within EDO gene families, providing new insights into the evolutionary mechanisms underlying microbial adaptation to complex pollutant mixtures (Terrón-González et al., 2016; Duarte et al., 2017; Brennerova et al., 2022). The EDO gene population was not dominated by canonical subfamilies. Phylogenetic analyses further revealed that most identified genes could not be assigned to previously defined EDO subfamilies. This extensive diversification underscores the role of environmental heterogeneity, particularly complex pollutant mixtures, as a major driver of metabolic versatility and gene evolution. Taken together, these findings demonstrate that EDO diversity in natural environments is far greater than previously recognized and represents a valuable genetic reservoir for biotechnological applications.

Functional metagenomic analyses based on enzyme function screening have further elucidated key evolutionary features of EDOs that enable microbial adaptation to PAH-enriched environments (Suenaga, 2015). Comparative studies of the I.2.G EDO subfamily derived from metagenomic datasets revealed that a small number of amino acid substitutions relative to the ancestral genotype can confer enhanced catalytic efficiency at the expense of thermal stability. This trade-off between catalytic activity and thermostability reflects adaptive optimization under selective pressures in contaminated habitats (Suenaga et al., 2007; Suenaga et al., 2009). The stability-function trade-off is a universal phenomenon during protein evolution that has been observed with completely different types of proteins, including enzymes, antibodies, and engineered binding scaffolds (Kurahashi et al., 2019; Teufl et al., 2022). Starting from a highly stable parent scaffold increases robustness against amino acid substitutions, allowing mutations that contribute to functional improvement while also causing destabilization, thereby expanding the space of potentially evolvable sequences (Tsuboyama et al., 2023).

## Industrial and biotechnological applications of metagenome-derived oxygenase

Beyond advancing evolutionary understanding, enzymatic machinery derived from environmental microorganisms for the degradation of PAHs presents significant opportunities for biocatalysis, industrial biotechnology, and bioremediation (Xiang et al., 2021). Aromatic ring monooxygenases, dioxygenases, and related oxygen-inserting enzymes catalyze highly selective hydroxylation reactions that are difficult to achieve using conventional chemical processes, particularly under mild reaction conditions and with minimal byproduct formation. Recent studies of *Pseudomonas* spp. have demonstrated that multicomponent aromatic ring-hydroxylating dioxygenases possess remarkable catalytic versatility, enabling accommodation of both low- and high molecular weight PAHs through flexible active site architectures (Yesankar et al., 2023). These properties make such enzymes attractive candidates for industrial oxidation chemistry and for the production of hydroxylated intermediates used in pharmaceuticals and fine chemicals.

Environmental microbiomes inhabiting PAH-rich environments, such as oil-contaminated soils, aquatic sediments, and wastewater treatment systems, represent a frontier of industrial relevance. These habitats impose strong selective pressures that enrich degradative microorganisms, resulting in the emergence of highly efficient oxidative catalysts (Verma and Satyanarayana, 2020; Mesbah, 2022). Various novel catechol 2,3-dioxygenases with excellent enzymatic properties were identified by the screening of metagenomic libraries (Sidhu et al., 2019). The enzyme with unique properties of optimal pH for acidic environments, salt tolerance, resistance to metal ions and organic solvents, and thermal stability, was obtained from PAH-contaminated soil suggesting promising candidate for bioremediation and industrial applications (Wang et al., 2025). Cytochrome P450 enzymes also play vital roles in the synthesis of industrially useful intermediates as well as in the degradation of polluted compounds, including PAHs (Petriti et al., 2025). Many P450 enzymes that catalyze the oxygenation of aromatic compounds have been identified from aromatic compound-degrading bacteria isolated from contaminated environments (Yang et al., 2023). Functional metagenomic surveys have revealed that such environments function as natural bioreactors in which diverse oxygenases, dehydrogenases, and ring-cleavage enzymes evolve, positioning environmental microbiomes as largely untapped reservoirs of industrially valuable biocatalysts. Metagenomic approaches therefore provide an effective and powerful framework for exploring and exploiting this enzymatic diversity.

## Future perspectives

Metagenomic analysis represents a powerful approach for elucidating the evolutionary dynamics of aromatic compound-degrading genes in natural ecosystems. By capturing a broad repertoire of gene homologs simultaneously, metagenomic approaches enable comparisons of sequence divergence, SNP patterns, and functional variation among coexisting enzymes, thereby providing high-resolution snapshots of the adaptive processes shaping catabolic pathways *in situ* (Suenaga, 2012). However, a single metagenome represents only a temporal snapshot of an ongoing evolutionary process. Accordingly, longitudinal metagenomic surveys offer substantial potential to track real-time shifts in allele frequencies, regulatory systems, and mobile genetic elements. Such time-series analyses would allow direct observation of how environmental pressures, including pollutant influx and nutrient availability, drive the continuous diversification and optimization of aromatic degradation gene systems and their microbial hosts.

Bacteria employ three principal evolutionary and organizational strategies for contributing to metabolic capability in environments contaminated with aromatic compounds: (i) gene mutation, (ii) horizontal gene transfer (HGT), and (iii) control of gene expression. The ability to utilize aromatic compounds often arises from mutations in genes encoding enzymes responsible for their transformation or degradation. Once acquired, these traits can be disseminated rapidly through HGT via mobile genetic elements, including plasmids, transposons, and integrative conjugative elements (ICEs). Transferred genes may be maintained on plasmids or integrated into the host chromosome through homologous recombination, thereby stabilizing the phenotype conferred by HGT. In addition, expression of genes can be optimized in the host strain. However, to understand the relationships and coordination among these three strategies, more accurate and careful argumentation is required.
